# Education, leisure activities and cognitive and functional ability of Alzheimer's disease patients: A follow-up study

**DOI:** 10.1590/S1980-57642013DN70200008

**Published:** 2013

**Authors:** Margarida Sobral, Constança Paúl

**Affiliations:** 1 MSc, Psychogeriatrics Service, Hospital Magalhães Lemos, Porto, Portugal.; 2 PhD, Research and Education Unit Aging, UNIFAI, Institute of Biomedical Sciences Abel Salazar, University of Porto, Portugal.

**Keywords:** aging, Alzheimer's disease, education, leisure activities

## Abstract

Education and participation in leisure activities appear to be highly relevant variables in Alzheimer's disease (AD) and usually form the basis of the Cognitive Reserve construct.

**OBJECTIVE:**

[A] To determine the association between education, cognitive and functional ability of AD patients; [B] To determine the association between participation in leisure activities and cognitive and functional ability of AD patients; [C] To evaluate the association of education and participation in leisure activities in the course of AD.

**METHODS:**

Functional and neuropsychological abilities of 120 outpatients with probable AD were evaluated at baseline, at 36 and 54 months. Data collected at baseline included socio-demographics, clinical variables, education and frequency of participation in leisure activities throughout life. All participants and/or caregivers answered the questionnaire, "Participation in leisure activities throughout life" while patients completed the MMSE, the Clinical Dementia Rating scale, neuropsychological tests from the Lisbon Screening for Dementia Assessment, Barthel Index and Lawton and Brody's Index.

**RESULTS:**

AD patients with higher levels of education achieved better results on cognitive tests. The participants with higher participation in leisure activities exhibited better results on cognitive and functional tests than those with lower participation. The disease progression was linear and progressed similarly regardless of the level of education of participants. However, the results suggest a slower disease progression in patients with a higher level of participation in leisure activities throughout their lives.

**CONCLUSION:**

AD patients with high education and high participation in leisure activities may benefit from a slower cognitive and functional decline after diagnosis of AD.

## Abstract (pt)

Escolaridade e participação em actividades de lazer parecem ser variáveis muito relevantes na doença de Alzheimer (DA) e normalmente usadas no construto Reserva Cognitiva.

**OBJETIVO:**

[A] Conhecer a associação entre escolaridade, capacidades cognitivas e funcionais de doentes com DA; [B] Conhecer a associação entre participação em actividades de lazer e capacidades cogntivas e funcionais de doentes com DA; [C] Avaliar a associação da escolaridade e participação em actividades de lazer no curso da DA.

**MÉTODOS:**

Foram avaliadas competências funcionais e neurospsicológicas de 120 doentes com provável DA na *baseline,* após 36 e 54 meses. Dados recolhidos na *baseline* incluíram variáveis sociodemográficas, clínicas, escolaridade e frequência da participação em actividades de lazer. Participantes ou/e cuidadores responderam ao questionário, "Participação em actividade de lazer ao longo da vida" e completaram o MMSE, CDR, provas da Bateria de Lisboa para Avaliação das Demências, Índice de Barthel e Índice Lawton e Brody.

**RESULTADOS:**

Doentes com DA com níveis mais elevados de escolaridade obtiveram melhores resultados nas provas cognitivas. Participantes com níveis mais elevados de participação em actividades de lazer exibiram melhores resultados nos testes cognitivos e funcionais do que aqueles com níveis mais baixos de participação. A progressão da doença foi linear e progrediu de forma semelhante relativamente ao nível de escolaridade. No entanto, resultados sugerem uma mais lenta progressão da doença relativamente aos doentes com níveis mais elevados de participação em actividades de lazer.

**CONCLUSÃO:**

Doentes com DA com elevada escolaridade e alta participação em actividade de lazer podem beneficiar de um mais lento declínio cognitivo e funcional após o diagnóstico da DA.

## INTRODUCTION

In the last few decades there has been an increase in the Portuguese elderly population. According to the 2011 Census, 19.1% of the population is over 65 years of age.^1^ At present, the Aging Index is 129, which means that for every 100 young people (population aged between 0-14 years) there are 129 elderly.^1^ The risk of dementia rises exponentially with age.^2-4^ However, studies have suggested that the prevalence of dementia increases exponentially up to 80 to 85 years, remaining stable or declining thereafter.^5,6^ Alzheimer's disease (AD) is the most common cause of dementia among elderly persons^3,7-9^ and is a progressive neurodegenerative disorder that is characterized by deterioration of cognitive and functional abilities and by a number of neuropsychiatric and behavioral symptoms. By contrast, in Japan and China Vascular Dementia accounts for almost 50% of all dementias.^10,11^

Cognitive Reserve (CR) is a hypothetical construct that has been used to explain cognitive aging^12^ and describes the capacity of the adult brain to tolerate the effects of this neurodegenerative process and to minimize the clinical manifestation of the pathology of dementia, and is probably the result of innate intelligence or life experience.^12,13^ The CR hypothesis suggests that individual differences in the ability to cope with AD pathology^12-14^ are consistent with the prediction that people with more reserve can cope with advancing AD pathology longer before it is expressed clinically.^13,15^ Reserve may explain why there is not a perfect relationship between brain pathology observed post-mortem and the severity of clinical conditions such as AD.^12^ Variables pertaining to lifetime experience (education, occupational attainment and leisure activities) are the most commonly used proxies for CR and help retain cognitive function in old age.

Concerning the relationships between education, work, hobbies and dementia, there is a line of research that indicates that individuals with higher education, occupational attainment or participation in leisure activities have a lower risk of developing dementia.^13,16-22^ Recently, Cindy Stern and Zachary Munn^21^ revealed evidence suggesting that active participation in cognitive leisure during mid or late life may be beneficial in preventing the risk of AD and other dementia in the elderly. Nevertheless, these authors showed that evidence is currently not strong enough to infer a direct causal relationship. Another line of research found no association between education and incident dementia^23,24^ and no association between occupation and incident AD in several population-based longitudinal studies.^25,26^

Research also explores the hypothesis that CR may introduce differences in the clinical course of AD.^27^ Some studies have shown that AD patients with higher education have more rapid cognitive decline^15,28^ than those with lower education, because at any level of clinical severity, the underlying progression is more advanced in patients with greater CR.^13,15^ Le Carret and colleagues^29^ confirmed that some cognitive processes, such as abstract thinking, decline more rapidly in AD patients with high education, whereas others seem to evolve more slowly compared to AD patients with low education. Research has indicated that high participation in leisure activities is associated with more rapid cognitive decline than those with lower participation in leisure activities.^30^ On the contrary Fritsch and colleagues^31^ concluded that education slows the rate of cognitive decline in individuals with AD. Treiber^32^ proved that increased engagement in cognitive leisure activities throughout late life was associated with slower deterioration in general cognitive ability in mild dementia, but its effects were no longer evident in more severe AD. Other studies have found no relationship between education and cognitive decline in the clinical course of AD.^33,34^

The results of studies regarding the association of education and participation in leisure activities and rates of cognitive decline seem to support CR, but the impact of this association on clinical outcomes remains unclear,^27^ namely the potential effect of CR on the course of AD.

The aims of this work were: [1] to determine the association between education and cognitive and functional ability of AD patients; [2] to determine the association between participation in leisure activities throughout life and cognitive and functional ability of AD patients; [3] to evaluate the association of education and participation in leisure activities throughout life in the course of AD.

## METHODS

**Participants.** This study included 120 outpatients diagnosed with probable AD, recruited at a psychiatric hospital from its psychogeriatrics service. The Psycho-geriatrics Service is designed to follow-up of patients with dementia, using an integrated multidisciplinary approach to diagnosing and managing dementia. Physical, neurological, neuropsychological and psychiatric examinations, neuroimaging and additional tests, including blood tests were used to distinguish between the various types of dementia. All patients fulfilled the criteria of both the Diagnostic and Statistical Manual of Mental Disorders, 4^th^ edition,^35^ and the National Institute for Neurological and Communicating Disorders and Stroke/AD and Related Disorders Association^36^ for probable AD. All participants were free of severe medical conditions other than those pertinent to the study. Normal or corrected levels of hearing and vision acuity were also ensured.

**Assessment instruments.** Data on participation in leisure activities was obtained from the questionnaire, "Participation in leisure activities throughout life", answered by the patient participants or/and their caregiver. The evaluation commenced by discriminating current and past activities. The present results show only activities undertaken by patients throughout life. The caregivers were asked to confirm the information about patient participation in leisure activities throughout life because low current participation in leisure activities of patients may be a consequence of cognitive decline. This tool included mental activities (reading books/newspapers, jigsaw puzzles), physical activities (walking or other sport), social activities (playing cards/board games, visiting friends or relatives), productive activities (housekeeping, babysitting, gardening, crocheting) and recreational activities (listening to the radio, watching television). Subjects reported the frequency of participation in leisure activities throughout life as "daily", "several days per week", "once a week", "two or three days per month", "monthly", or "never or less than once a month". Participants were asked if they regularly engaged in any other particular activities, to specify which types of activities and to report the frequency of participation in leisure activities throughout life. The instrument had a total score and classified respondents into three categories (low, medium and high participation in leisure activities throughout life). The questionnaire "participation in leisure activities throughout life", has 17 questions and for each question subjects received 5 points for "daily", 4 points for "several days per week", 3 points for "once a week", 2 points for "two or three days per month", 1 point for "monthly" and 0 points for "never or less than once a month". Total score ranges from 0 to 85 points. Subjects were classified as having low participation in leisure activities if they scored below 20, medium for scores between 20 and 25 and high for scores higher than 25.

All participants were administered the Mini-Mental State Examination (MMSE)^37^ for cognitive screening, as well as the Clinical Dementia Rating (CDR) scale^38^ which classifies dementia under 3 stages of severity as a function of overall cognitive and functional impairment. The Portuguese version of the MMSE from Guerreiro et al.^39^ was applied. The normative cut-off education-adjusted values for the Portuguese population were used.^39^ Subjects were expected to score above 27 for >11 years of education, ≤22 for 1-11 years of education or ≤15 if they were illiterate. The CDR determines the impairment associated to dementia, through parameters such as memory, orientation, judgment and problem solving, community affairs, home and hobbies and personal care. The overall CDR score is obtained by a standard algorithm to stage the patient's level of impairment: 0, no impairment; 0.5, very mild impairment; 1, mild dementia; 2, moderate dementia; and 3, severe dementia. The scale is administered to both patient and informant through a semi-structured interview.

AD patients were also assessed with the Lisbon Battery for Assessment of Dementia (BLAD),^40^ aimed at evaluating cognitive abilities within the realm of language (understanding of simple instructions, writing, object naming and identification), orienting, calculating, memory, attention, executive function, motor and grapho-motor initiative, and visuospatial ability.

Functional abilities were assessed with two activities of daily living scales: Barthel's Index, addressing basic activities of daily living such as grooming, eating, bathing, dressing, and mobility, along with Lawton and Brody's Index, targeting instrumental activities (e.g. managing money, using the telephone). The Barthel Index has a total possible score ranging from 0 to 100 (fully independent) whereas Lawton and Brody's Index has a total possible scores ranging from 8 (independent) to 30 (completely dependent). Socioeconomic status was evaluated according to the Graffar Index.^41^

**Procedure and statistical design.** Data collected at baseline comprised socio-demographic and clinical variables including age, gender, marital status, retirement status and variables used to represent CR (educational level and frequency of participation in leisure activities). Each participant underwent a standard evaluation, including medical history, physical examination, laboratory tests and a neuro-imaging scan (computed axial tomography). All AD patients were evaluated with the MMSE and CDR for recruitment at the first consultation of multidisciplinary assessment. All patients were submitted to a functional and neuropsychological evaluation. Functional and neuropsychological abilities of patients were revaluated at 36 and 54 Specific neuropsychological domains were examined including memory, language, attention, visuospatial ability and executive functioning. At follow-up^1^, 0% (n=0) of the initial sample dropped out of the study, but after 54 months, 26.66% (n=32) of the initial sample had dropped out. The drop-outs were due to refusal and to deaths in the course of 54 months.

In this study, two variables were hypothesized to represent the CR construct at baseline: education (as defined in terms of level of educational attainment) and frequency of participation in leisure activities (as defined in terms of low, medium and high leisure activities). General exploratory analyses were conducted to determine sample characteristics. The *t-*tests were used to compare neuropsychological and functional measures between baseline, follow-up 1 (F1) and follow-up 2 (F2). Pearson's correlations were used to examine the association between education and leisure activities, the association between education level and social class, and the association between leisure activities and social class.

An ANOVA mixed effects model was employed to study the effects of age at baseline, gender, CR measured by education (with 4 levels: illiterate (cannot read or write), reading and writing (read and write, but without formal education or with 1-3 years of education), 4 years (4 years of education) and ">4 years" (more than 4 years of education), and participation in leisure activities (with 3 levels: low, medium and high), on neuropsychological performance at baseline, at 36 and 54 months. A complementary set analysis was conducted using the disease progression index to examine the influence of CR on cognitive and functional decline over 36 (3 years) and 54 months (4.5 years). The disease progression index (DPI) was calculated for 3 years and for 4.5 years. The cognitive DPI was calculated as the difference between cognitive scores at baseline and F1 after 3 years, and between baseline and F2 after 4.5 years. The functional DPI was calculated as the difference between functional scores at baseline and F1 after 3 years, and between baseline and F2 after 4.5 years.

Statistical analyses were conducted using the Statistical Package for Social Sciences version 18.0 (SPSS).

## RESULTS

For the present analyses, data from 120 patients with dementia at baseline was used: baseline mean age of the population was 78.15 (SD 6.69 years) and educational level was 4 years. Males and females differed significantly in level of education (5.92 years for males; 3.07 years for females; t=4.491 p<.001). At baseline, 86.7% were classified as CDR=1 (mild dementia) and 13.3% as CDR=2 (moderate dementia). After 36 months 45% were classified as CDR=1, 43.3% as CDR=2 and 11.7% as CDR=3. After 54 months 36.4% were classified as CDR=1, 42% as CDR=2 and 21.6% as CDR=3 (severe dementia). Table 1 shows the baseline sociodemographic and clinical characteristics of AD participants.

**Table 1:** Demographic and clinical characteristics of AD patients.

Demographic and clinical characteristics		AD patients (n=120)
Men (%) (n)		31.7 (38)
Women (%) (n)		68.3 (82)
Age at baseline (mean) (SD)		78.15 (6.69)
Married (%) (n)		60.8 (73)
Widower (%) (n)		35 (42)
Single (%) (n)		3.3 (4)
Divorced (%) (n)		0.8 (1)
Educational level (mean)(SD)		4 (3.48)
Illiterate (%) (n)		17.5 (21)
Reading and writing (%) (n)		28.3 (34)
4 years (%) (n)		39.2 (47)
9 years (%) (n)		8.3 (10)
11 years (%) (n)		4.2 (5)
>11 years (%) (n)		2.5 (3)
Work _Retired (%) (n)		97.5 (117)
Portuguese nationality (%) (n)		100 (120)
Years of disease (mean)(SD)		4.93 (2.68)
Social Class (Graffar) n (%)	I (high) and II (medium/high)	9 (7.5)
	III (medium)	39 (32.5)
	IV (medium/low) and V (low)	72 (60.0)
Live alone	Yes (%) (n)	10.8 (13)
	No (%) (n)	89.2 (107)
Live with his wife/her husband	Yes (%) (n)	60 (72)
	No (%) (n)	40 (48)
Attend day care	Yes (%) (n)	20 (24)
	No (%) (n)	80 (96)
Live in a nursing home	Yes (%) (n)	3.3 (4)
	No (%) (n)	96.7 (116)
CDR = 1 at baseline (%) (n)		86.7 (104)
CDR = 2 at baseline (%) (n)		13.3 (16)

The neuropsychological test mean scores and functional scores at baseline were compared with those collected at 36 months and at 54 months in AD patients. Tests revealed a significant decline in MMSE score (p<.001), verbal memory with interference (BLAD), digit span (BLAD), proverbs (BLAD), information (BLAD), calculation (BLAD), orientation (BLAD), motor initiative and language (repetition of words) (BLAD) between scores at baseline and F1 and also between scores at F1 and F2. Statistically significant difference between baseline and F1 were observed in functional status, Barthel's Index (p<.001) and Lawton and Brody's Index (p<.001) and between F1 and F2. There was a significant decline in cognitive and functional performance over time across all domains. Data analysis showed cognitive and functional decline over the 54-month period. The results of this study are in agreement with knowledge about AD, a disease characterized by progressive cognitive deterioration, together with declining activities of daily living.

Pearson's correlation (r=.316) was significant at the .01 level (2-tailed) between education and participation in leisure activities. Positive correlations were found between educational level and socioeconomic class and between leisure activities and socioeconomic class.

According to the ANOVA mixed effects model for disease progression, as measured by the MMSE, there was no significant difference between men and women (p=.428) or across the age span (p=.725) on cognitive performance.

**Figure 1:**
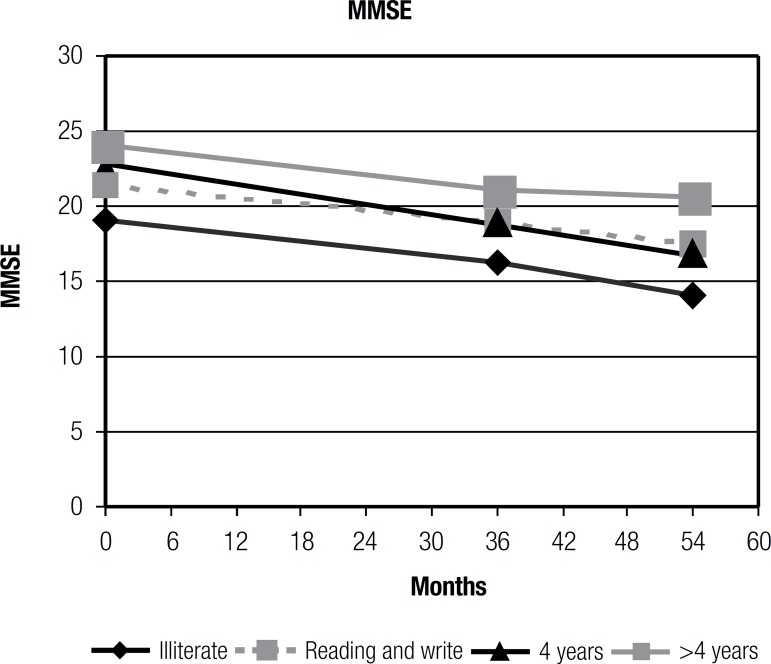
Patterns of MMSE obtained for different educational levels.

**Education.** Figure 1 shows the patterns of MMSE obtained for different educational levels.

The "> 4 years" group (more than 4 years of education) obtained the highest scores on the MMSE. The AD patients with higher levels of education achieved better results on the MMSE. The participants with higher educational levels had higher scores on cognitive tests than elderly with lower educational levels.

The mean Barthel Index of participants with "<4 years" group (less than 4 years of education) at baseline was 96.25 and with ">4 years" group was 99.55, whereas the mean Barthel Index of AD patients with "<4 years" group at F2 was 83.29 and with ">4 years" group was 84.12. The mean Lawton and Brody Index of participants with "<4 years" group at baseline was 16.91 and with ">4 years" group was 18.14, while the Lawton and Brody's Index mean scores of participants with "<4 years" group at baseline versus F2 was 16.91/23.78 and with ">4 years" group was 18.05/25.65.

Pearson's correlation was significant at the .01 level (2-tailed) between education and MMSE (at baseline, F1 and F2), but was not correlated with the functional scales.

According to the ANOVA mixed effects model for disease progression, as measured by the MMSE, there was no significant difference between educational level (with 4 levels: illiterate, reading and writing, 4 years and ">4 years") (p=.338) on cognitive performance. The participants with greater years of education did not decline at significantly faster than individuals with fewer years of education. The ">4 years" group had a DPI, assessed with the MMSE, of 0.91 (36 months) and 0.99 (54 months). The "=4 years" group (4 years of education), assessed with the MMSE, had a DPI of 1.31 (36 months) and 1.32 (54 months). The "<4 years" group, assessed with the MMSE, had a DPI of 1.03 (36 months) and 0.84 (54 months). In this study, results of disease progression were found to be very similar for all levels of education.

**Participation in leisure activities.** In this study, 68.3% of the participants had low participation in leisure activities throughout their lives, 20.8% medium and 10.8% high participation. At baseline, for the "low leisure activities" group, 82.92% of patients were classified as CDR=1 and 17% as CDR=2. In the "medium leisure activities" group, 92% of individuals were classified as CDR=1 and 8% as CDR=2. In the "high leisure activities" group, 100% of individual were classified as CDR=1. At 36 months in the "low leisure activities" group, 36.58% of individuals were classified as CDR=1, 47.56% as CDR=2 and 15.85% as CDR=3. In the "medium leisure activities" group, 52% of participants were classified as CDR=1, 44% as CDR=2 and 4% as CDR=3. In the "high leisure activities" group, 84.61% of elderly participants were classified as CDR=1 and 5.38% as CDR=2. At 54 months in the "low leisure activities" group, 28.84% of participants were classified as CDR=1, 42.3% as CDR=2 and 28.84% as CDR=3. In the "medium leisure activities" group, 37.5% of individuals were classified as CDR=1, 50% as CDR=2 and 12.5% as CDR=3. In the "high leisure activities" group, 66.66% of participants were classified as CDR=1, 25% as CDR=2 and 8.33% as CDR=3.

Pearson's correlation was significant at the .01 level (2-tailed) between participation in leisure activities and MMSE (at baseline, F1 and F2), but was not correlated with the functional scales.

**Figure 2:**
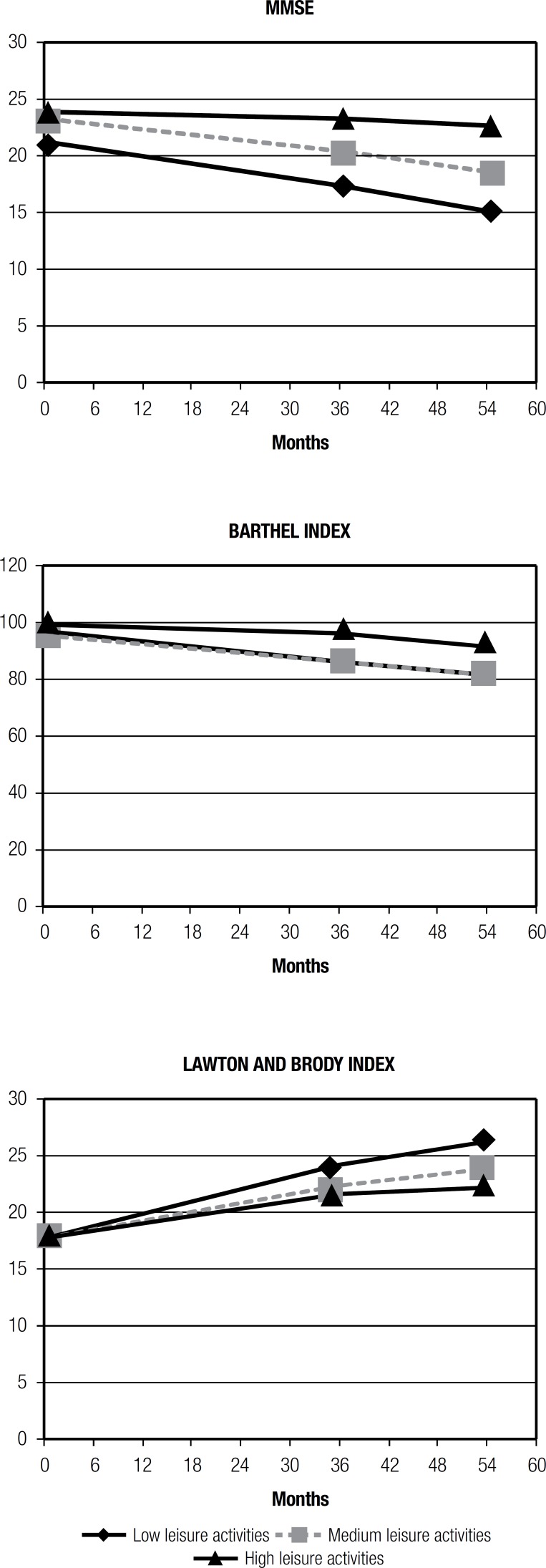
MMSE, Barthel Index, Lawton and Brody Index and participation in leisure activities

Figure 2 shows that the participants with higher participation in leisure activities exhibited better results on cognitive and functional tests than those with lower participation in leisure activities throughout life. The cognitive and functional decline appears to be more gradual for high participation in leisure activities.

According to the ANOVA mixed effects model for disease progression, as measured by the MMSE, a significant difference was found between participation in leisure activities (low, medium and high) (p=0.012) and cognitive performance. The participants with high scores for participation in leisure activities, declined (global cognitive) at a significantly slower rate than participants with low scores. At F1, the average difference in rate of decline, DPI, on the MMSE, for high versus low leisure activities was 0.15/1.25 and at F2 was 0.24/1.34. The functional decline was found to be more gradual for high leisure activities group. On average, the difference in rate of decline, DPI, on the Barthel Index for high versus low leisure activities at 36 months was 1.02/3.45 and at 54 months was 1.76/3.31. These results suggest a slower disease progression for patients with a higher level of participation in leisure activities.

## DISCUSSION

Our first objective was to determine the association between education and cognitive and functional ability of AD patients, and this study showed that AD patients with higher levels of education achieved better results on cognitive tests and better mean scores on Barthel's Index, but not reaching statistically significance at baseline. In Portugal, most of the old people have a low level of education. In 2010, 34.81% of the resident population aged 65 years and over did not have education beyond the fourth year of school and 46.94% of the resident population aged 65 years and over had 4 years of education.^42^ In this study, the participants had an average level of education of 4 years and only 18 patients had more than 4 years of education. Many of the participants may not have achieved a level of higher education, regardless of their intelligence, for socio-economic reasons. In normal aging, it is generally accepted that greater education is associated with better cognitive test performance in older adults. Aevarsson and Skoog^43^ showed that higher education was related to higher scores on the MMSE at age 85 while other authors^44^ showed that low education was associated with poor cognitive performance. Albert and colleagues^45^ found that persons with higher education attainment exhibit less cognitive decline with advancing age. Our findings are consistent with several studies showing that persons with higher education perform better on cognitive tests than less educated individuals.^46^

Our second objective was to determine the association between participation in leisure activities throughout life and cognitive and functional ability of AD patients, and we observed that the elderly participants with higher participation in leisure activities exhibited better results on cognitive and functional tests than those with lower participation in leisure activities throughout life. Many studies have investigated the association between level of participation in activities and performance on cognitive tests in healthy adults.^13,47^ High participation in leisure activities has also been associated with better outcomes on cognitive tests. Scarmeas and colleagues^16^ proved that subjects with better baseline cognitive performance had higher leisure activity scores. Our results confirmed findings of a previous research study^22^ demonstrating that leisure activities (social, mental, and physical) were beneficial to cognition and also support another study^48^ reporting that cognitive stimulation with leisure activities had a positive association with cognitive and functional abilities of AD patients. Leisure activities can be broadly divided into cognitive and physical activities and some studies^16,49^ have included participation in cognitive and physical leisure activities since both contribute positively. This study included participation either in cognitive or physical leisure activities, where both activities were significantly correlated in this research.

Our third objective was to evaluate the association of education and participation in leisure activities throughout life in the course of AD. Results showed slower disease progression for AD patients that had a higher level of participation in leisure activities. The low education together with low level of participation in the present sample may hinder our results because most participants shared the same status of low educational level. The intellectual challenges experienced during life, accumulate reserve and allow cognitive function to be maintained in old age.^50^ CR has been conceptualized as a dynamic construct. Researchers have suggested that variables that reflect lifetime experiences, education and participation in leisure activities, were proxies of CR and can help to mitigate the impact of pathology on the clinical expression of dementia. Numerous studies have indicated that CR (education and participation in leisure activities) delay the onset of dementia,^16-19^ but that after onset, CR is linked to more rapid progression.^15,30^ Nevertheless, a few other studies have shown that high education slows the rate of cognitive decline in persons with dementia^31^ and that high participation in leisure activities is associated with slower deterioration in general cognitive ability.^32,48^ Several studies have failed to find any relation between education and cognitive decline in dementia.^33^ In line with our results, Treiber^32^ proved that increased engagement in cognitive leisure activities throughout late life was associated with slower deterioration in general cognitive ability in mild dementia whereas Katzman and colleagues^33^ found no relationship between education and cognitive decline in the clinical course of AD.

The main limitation of this study was the small sample size where the number of participants did not allow stratification by severity of disease for analysis of the progression of each stage of dementia; the drop-outs at follow-up affected the conclusion. Nevertheless, because of the potential value of the results they should be considered for future research.

In conclusion, considering our objectives of determining the association between education and participation in leisure activities and cognitive and functional results of AD patients, this study showed that AD patients with higher levels of education achieved better results on cognitive tests. The elderly participants with higher participation in leisure activities exhibited better results on cognitive and functional tests than those with lower participation in leisure activities throughout life.

Concerning the last aim of this study, namely, to evaluate the association of education and participation in leisure activities in the course of AD, data analysis showed a cognitive and functional decline during the 54-month period. The disease progression was linear and progressed similarly regardless of the level of education of participants. However, it should be reiterated that most of the elderly participants had a low level of education. The participants had an average level of 4 years' education. This work showed that high participation in leisure activities and high education were not associated with a more rapid cognitive decline compared with lower participation in leisure activities and lower education. The results suggest slower disease progression for patients with a higher level of participation in leisure activities throughout their lives. AD patients with high education and high participation in leisure activities may gain benefits after diagnosis of AD, through slowing of cognitive and functional decline.

Future studies could follow a sample of elderly patients with more years of education (≥ 11 years) to track disease progress.

## Figures and Tables

**Table 2:** Comparison between neuropsychological (MMSE, BLAD) and functional functions (Barthel Index plus Lawton and Brody's Index) at baseline, follow-up 1 and follow-up 2.

Neuropsychological and Functional Assessment	BaselineMean (SD)	Follow-up 1Mean (SD)	p1(Baseline x F1)	Follow-up 2Mean (SD)	p2(F1 x F 2)
Barthel Index	97.04 (8.78)	87.63(15.20)	< 0.001	83.35 (17.59)	< 0.001
Lawton and Brody's Index	17.75 (5.34)	23.53 (5.65)	< 0.001	24.99 (5.15)	< 0.001
MMSE	21.96 (4.22)	18.73 (5.65)	< 0.001	17.16 (6.56)	< 0.001
**Lisbon Battery for Assessment of Dementia (BLAD)**					
Writing	1.49 (0.86)	1.37 (0.91)	0.033	1.25 (0.96)	0.006
Reading	1.51 (0.85)	1.39 (0.91)	0.027	1.30 (0.94)	0.027
Language_Appointment	7.00 (0.000)	6.61 (1.52)	0.007	6.02 (2.39)	0.026
Language_Identification object	5.00 (0.00)	4.71 (1.15)	0.008	4.49 (1.50)	0.374
Language_Repetition of words	10.28 (1.36)	9.48 (2.40)	< 0.001	8.90 (3.08)	0.004
Language_Simple instructions	3.97 (0.36)	3.72 (0.98)	0.016	3.48 (1.31)	0.042
Calculation	8.26 (4.47)	6.46 (4.76)	< 0.001	5.06 (4.71)	< 0.001
Orientation	8.83 (3.07)	6.01 (3.13)	< 0.001	4.85 (3.29)	< 0.001
Proverbs	4.66 (1.88)	3.91 (1.39)	< 0.001	3.52 (1.06)	< 0.001
Graphomotor initiative	1.10 (1.02)	0.75 (1.12)	0.002	0.49 (0.66)	0.013
Motor initiative	1.83 (1.12)	1.32 (1.20)	< 0.001	1,01 (1,14)	0.002
Semantic Fluency	9.02 (3.46)	6.91 (3.82)	< 0.001	6.22 (3.93)	0.195
Digit Span	7.63 (2.15)	6.59 (2.68)	< 0.001	5.60 (3.93)	0.008
Digit Span Forward	5.28 (1.28)	4.67 (1.74)	< 0.001	4.03 (2.00)	0.060
Digit Span Backward	2.37 (1.37)	1.94 (1.49)	0.012	1.63 (1.67)	0.272
Verbal memory with interference	6.54 (3.07)	4.89 (3.42)	< 0.001	3.87 (3.36)	0.001
Information	8.51 (4.73)	6.76 (4.63)	< 0.001	5.50 (4.49)	0.001

p1: Indicates statistically significant changes between baseline and follow-up 1 on paired t-test. p2 :Indicates statistically significant changes between baseline and follow-up 2 on paired t-test.

## References

[1] Instituto Nacional de Estatística (2011). Censos 2011 - Resultados Provisórios [INE web site].

[2] Fratiglioni L, Launer LJ, Andersen K (2000). Incidence of dementia and major subtypes in Europe: A
collaborative study of population-based cohorts. Neurologic Diseases in the
Elderly Research Group. Neurology.

[3] Lopes MA, Bottino CMC (2002). Prevalência de demência em diversas regiões
do Mundo. Análise dos estudos epidemiológicos de 1994 a 2000. Arq
Neuropsiquiatr.

[4] Ziegler-Graham K, Brookmever R, Johnson E, Arrighi HM (2008). Worldwide variation in the doubling time of Alzheimer's disease
incidence rates. Alzheimers Dis.

[5] Breitner JC, Wyse BW, Anthony JC (1999). APOE-epsilon4 count predicts age when prevalence of AD increases,
then declines: The Cache County Study. Neurology.

[6] Ritchie K, Kildea D, Robine JM (1992). The relationship between age and the prevalence of senile
dementiia: a meta-analysis of recente data. Int J Epidemiol.

[7] Cummings JL (2004). Alzheimer's Disease. New Engl J Med.

[8] Berr C, Wancata J, Ritchie K (2005). Prevalence of dementia in elderly in Europe. Eur Neuropsychopharmacol.

[9] Jalbert JJ, Daiello LA, Lapane K (2008). Dementia of the Alzheimer Type. Epidemiol Rev.

[10] Sachdev P (1999). Vascular Cognitive disorder. Int J Geriatric Psychiatr.

[11] Ikeda M, Hokoishi K, Maki N (2001). Increased prevalence of vascular dementia in Japan: a
community-based epidemiological study. Neurology.

[12] Siedlecki K, Stern Y, Reuben A, Sacco RL, Elkind MSV, Wright C (2009). Construct validity of cognitive reserve in multiethnic cohort:
The Northen Manhattan Study. J Int Neuropsychol Soc.

[13] Scarmeas N, Stern Y (2003). Cognitive Reserve and Lifestyle. J Clin Exp Neuropsychol.

[14] Stern Y (2002). What is cognitive reserve?. Theory and research application of the reserve concept. J Int
Neuropsychol Soc.

[15] Scarmeas N, Albert SM, Manly JJ, Stern Y (2006). Education and rates of cognitive decline in incident Alzheimer's
disease. J Neurol Neurosurg Psychiatry.

[16] Scarmeas N, Levy MD, Tang M-X, Manly J, Stern Y (2001). Influence of leisure activity on the incidence of Alzheimer's
Disease. Neurology.

[17] Verghese J, Lipton RB, Katz MJ (2003). Leisure Activities and the Risk of Dementia in the
Elderly. New Eng J Med.

[18] Scarmeas N, Stern Y (2004). Cognitive Reserve: Implications for Diagnosis and Prevention of
Alzheimer's Disease. Curr Neurol Neurosci Rep.

[19] Karp A, Paillard-Borg S, Wang H-X, Silverstein M, Winblad B, Fratiglioni L (2006). Mental, Physical and Social Components in Leisure Activities
Equally Contribute to Decrease Dementia Risk. Dement Geriatr Cogn Disord.

[20] Paillard-Borg S, Fratiglioni L, Winblad B, Wang H-X (2009). Leisure Activities in Late Life in Relation to Dementia Risk:
Principal Component Analysis. Dement Geriatr Cogn Disord.

[21] Stern C, Munn Z (2010). Cognitive leisure activities and their role in preventing
dementia: a systematic review. Int J Evid Based Healthc.

[22] Fratiglioni L, Paillard-Borg S, Winblad B (2004). An active and socially integrated lifestyle in late life might
protect against dementia. Lancet Neurol.

[23] Paykel ES, Brayne C, Huppert FA, Gill C, Barkley C, Gehlhaar E (1994). Incidence of dementia in a population older than 75 years in the
United Kingdon. Arch Gen Psychiatry.

[24] Graves AB, Larson EB, Edland SD (1996). Prevalence of dementia and its subtypes in the Japanese American
population of King County, Washington State: The Kame
Project. Am J Epidemiol.

[25] Jorm AF, Rodgers B, Henderson AS, Korten AE, Jacomb PA, Christensen H (1998). Occupation type as a predictor of cognitive decline and dementia
in old age. Age Ageing.

[26] Helmer C, Letenneur L, Rouch I (2001). Occupation during life and risk of dementia in French elderly
community residents. Neurol Neurosurg Psychiatry.

[27] Beelen MJ (2009). Cognitive Reserve in Alzheimer's Disease: Implications for
detection and prevention. JLGH.

[28] Wilson RS, Li Y, Aggarwal NT (2004). Education and the course of cognitive decline in
Alzheimer. Neurology.

[29] Le Carret N, Auriacombe S, Letenneur L, Bergua V, Dartigues JF, Fabrigoule C (2005). Influence of education on the pattern of cognitive deterioration
in AD patients: the cognitive reserve hypothesis. Brain Cogn.

[30] Helzner E, Scarmeas N, Cosentino S, Portet F, Stern Y (2007). Leisure Activity and Cognitive Decline in Incident Alzheimer
Disease. Arch Neurol.

[31] Fritsch T, McClendon MJ, Smyth K, Ogrocki PK (2002). Effects of educational attainment and occupational status on
cognitive and functional decline in person with Alzheimer type
dementia. Int Psychogeriatrics.

[32] Treiber K Relationship of Cognitive Reserve and Decline in Alzheimer's Disease. A
Population Study.

[33] Katzman R, Brown T, Thal LJ, Fuld PA, Aronson M, Butters N (1988). Comparison of rate of annual change of mental status score in
four independent studies of patients with Alzheimer's
disease. Ann Neurol.

[34] Filley CM, Brownell HH, Albert ML (1985). Education provides no protection against Alzheimer's
disease. Neurology.

[35] American Psychiatric Association (2000). Diagnostic and Statistical Manual of Mental Disorders
(DSM-IV-TR).

[36] McKhann G, Drachman D, Folstein M, Katzman R, Price D, Stadlan EM (1984). Clinical diagnosis of Alzheimer's disease: report of the
NINCDS-ADRDA Work Group under the auspices of Department of Health and Human
Services Task Force on Alzheimer's Disease. Neurology.

[37] Folstein MF, Folstein SE, McHugh R (1975). Mini-Mental State: A practical method for grading the cognitive
state for patients for the clinician. J Psychiatr Res.

[38] Hughes CP, Berg L, Danzinger LW, Coben LA, Martin RL (1982). A new clinical scale for the staging of dementia. Br J Psychiatry.

[39] Guerreiro MS, Botelho MA, Leitão O, Castro Caldas A, Garcia C (1994). Avaliação Breve do Estado Mental. Adaptação
Portuguesa do Mini Mental State Examination (MMSE) (Folstein, Folstein,
McHugh, 1975).

[40] Garcia C (1984). Doença de Alzheimer, problemas do diagnóstico
clínico [Alzheimer'disease, difficulties in clinical
diagnosis].

[41] Graffar M (1956). Une méthode de classification sociale
d'échantillons de population. Courier.

[42] (2011). Pordata [database online].

[43] Aevarsson O, Skoog I (2000). A Longitudinal Population Study of the Mini-Mental State
Examination in the Very Old: Relation to Dementia and
Education. Dement Geriatr Cogn Disord.

[44] Kilander L, Nyman H, Boberg M, Lithell H (1997). Cognitive function, vascular risk factors and
education. A cross-sectional study based on a cohort of 70-year- old men. J Intern
Med.

[45] Albert MS, Jones K, Savage CR (1995). Predictors of cognitive change in older persons: MacArthur
studies of successful aging. Psychol Aging.

[46] Ganguli M, Ratcliff G, Huff FJ, Kancel MJ (1991). Effects of age, gender, and education on cognitive tests in a
rurural elderly community sample: norms from the Monongahela Valley
Independent Elders Survey. Neuroepidemiology.

[47] James BD, Wilson RS, Barnes LL, Bennett DA (2011). Late-life Social Activity and Cognitive Decline in old
Age. J Int Neuropsychol Soc.

[48] Teiber KA, Carlson MC, Corcoran C (2011). Cognitive stimulation and cognitive and functional decline in
Alzheimer's Disease: The cache county dementia progression
Study. J Gerontol B Psychol Sci Soc Sci.

[49] Friedland RP, Fritsch T, Smyth KA (2001). Patients with Alzheimer's disease have reduced activities in
midlife compared with healthy control-group members. Proc Natl Acad Sci USA.

[50] Staff TR, Murray AD, Deary IJ, Whalley LJ (2004). What provides cerebral reserve?. Brain.

